# Systemic Overexpression of TNFα-converting Enzyme Does Not Lead to Enhanced Shedding Activity *In Vivo*

**DOI:** 10.1371/journal.pone.0054412

**Published:** 2013-01-14

**Authors:** Masaki Yoda, Tokuhiro Kimura, Takahide Tohmonda, Hideo Morioka, Morio Matsumoto, Yasunori Okada, Yoshiaki Toyama, Keisuke Horiuchi

**Affiliations:** 1 Anti-aging Orthopedic Research, School of Medicine, Keio University, Tokyo, Japan; 2 Department of Orthopedic Surgery, School of Medicine, Keio University, Tokyo, Japan; 3 Pathology, School of Medicine, Keio University, Tokyo, Japan; University Paris Sud, France

## Abstract

TNFα-converting enzyme (TACE/ADAM17) is a membrane-bound proteolytic enzyme with a diverse set of target molecules. Most importantly, TACE is indispensable for the release and activation of pro-TNFα and the ligands for epidermal growth factor receptor *in vivo*. Previous studies suggested that the overproduction of TACE is causally related to the pathogenesis of inflammatory diseases and cancers. To test this hypothesis, we generated a transgenic line in which the transcription of exogenous *Tace* is driven by a CAG promoter. The *Tace*-transgenic mice were viable and exhibited no overt defects, and the quantitative RT-PCR and Western blot analyses confirmed that the transgenically introduced *Tace* gene was highly expressed in all of the tissues examined. The *Tace*-transgenic mice were further crossed with *Tace^−/+^* mice to abrogate the endogenous TACE expression, and the *Tace*-transgenic mice lacking endogenous *Tace* gene were also viable without any apparent defects. Furthermore, there was no difference in the serum TNFα levels after lipopolysaccharide injection between the transgenic mice and control littermates. These observations indicate that TACE activity is not necessarily dependent on transcriptional regulation and that excess TACE does not necessarily result in aberrant proteolytic activity *in vivo*.

## Introduction

Various membrane-bound molecules are subjected to proteolytic cleavage at the cell surface. This proteolytic activity, also referred to as “ectodomain shedding,” plays essential roles in the functional regulation of membrane-bound molecules. Previous studies revealed that the TNFα-converting enzyme (TACE), also known as A Disintegrin and Metalloprotease 17 (ADAM17), is one of the most critical proteolytic enzymes involved in ectodomain shedding *in vivo* [1]–[3]. TACE was originally identified as a converting enzyme responsible for the release of membrane-anchored pro-TNFα from cell surface [4], [5]. However, subsequent studies found an exceptionally large number of target molecules for TACE, including the ligands for epidermal growth factor receptor (EGFR), TNF receptor (TNFR)-1 and -2, CD62L/L-selectin, and vascular growth factor receptor 2 [6]–[9]. The importance of the functions of TACE *in vivo* was further underscored by the observation that mice lacking TACE die perinatally with a highly complex phenotype [6], [9]. Importantly, studies in TACE mutant mice revealed that TACE is indispensable for the functional activation of the pro-TNFα and EGFR ligands *in vivo* [6], [9].

There is evidence suggesting that the overexpression of TACE is causally related to the pathogenesis of various disorders [10], [11]. The overproduction of TACE has been reported in cancers, including breast cancer, colon carcinoma, lung cancer, and hepatocellular carcinoma [12]–[16], and inflammatory diseases, such as Sjögren's syndrome, osteoarthritis, and rheumatoid arthritis [17]–[20]. In these cases, TACE overexpression was suggested to promote pathogenesis through the excess cleavage of TACE substrates, such as EGFR ligands and TNFα. These observations, in turn, indicate that TACE activity is regulated, at least in part, at the transcriptional level *in vivo*.

TACE is initially produced as a proteolytically inactive pro-form and is processed by a furin protease in the secretory pathway to produce the mature enzyme [21]. However, the mechanisms underlying the functional activation of TACE remain elusive. TACE can be activated by various stimuli *in vitro*, including growth factors, phorbol esters, osmotic pressure, ultraviolet irradiation, and cholesterol deprivation, without changing the amount of its mature form [22]–[25]. Several studies have suggested that the phosphorylation of the cytoplasmic domain is involved in activation of TACE [26]–[29]; however, other findings have failed to support this hypothesis [22], [25]. Furthermore, a recent study showed that tissue inhibitor of metalloprotease 3 (TIMP3), an endogenous inhibitor of MMPs and TACE, directly binds to TACE and thereby regulates its proteolytic activity [29]. However, it has been shown that in cells lacking TIMP3, TACE can still be stimulated by phorbol esters [25], raising questions about the true nature of TACE activation.

To address these issues, we generated a transgenic line of mice that express TACE under the control of a CAG promoter and found that the mutant mice were viable and exhibited no apparent defects. A higher amount of TACE transcripts and mature protein in the mutant mice tissues were confirmed by quantitative RT-PCR and Western blotting, respectively. The expression levels of the TIMP3 transcripts were not affected by the introduction of the *Tace*-transgene in any of the tissues examined. This study, therefore, shows that the overproduction of TACE does not necessarily result in the hyperactivation of shedding activity and suggests that the level of TACE activity cannot be explained solely by its level of transcription or the amount of the mature form of the protein.

## Materials and Methods

### Generation of *Tace*-transgenic mice

**Figure 1:**
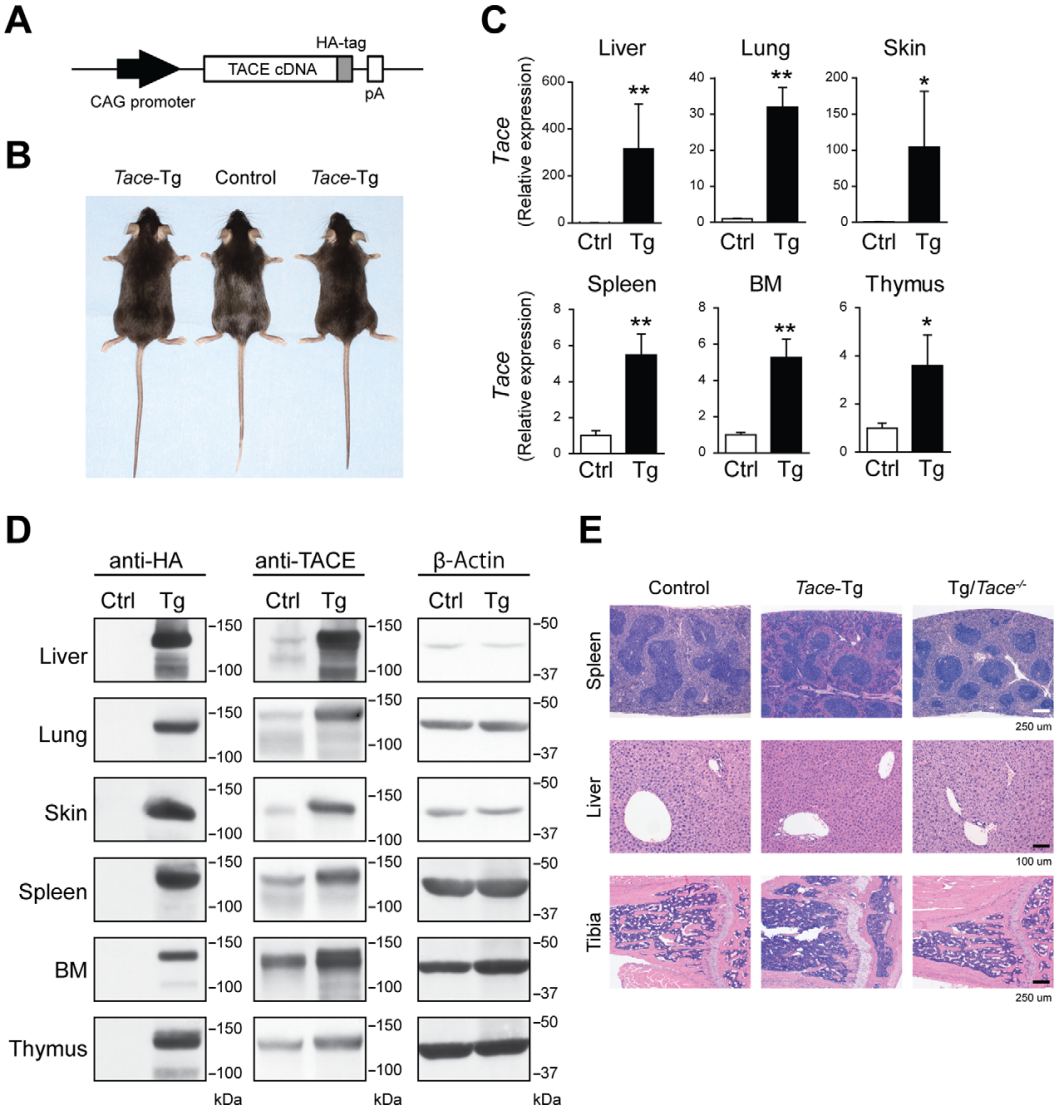
The systemic overexpression of TACE causes no overt defects. (A) A schematic of the transgene construct. pA, polyadenylation signal sequence. (B) Gross morphology of the 8-week-old control (Ctrl) and *Tace*-Tg (Tg) mice. (C) Quantitative RT-PCR analysis of *Tace* expression in the liver, lung, skin, spleen, bone marrow cells (BM), and thymus from 8-week-old control (Ctrl) and *Tace*-Tg (Tg) mice. The expression level of *Tace* in each organ of the control mice is set to 1. Bars, S.D. *p<0.05. **p<0.005. (D) Western blot analysis using anti-HA, anti-TACE, and anti-β-Actin antibodies. (E) Hematoxylin and eosin-stained sections of the spleen, liver, and tibia from 8-week-old control, *Tace*-Tg, and *Tace*-tg/*Tace^−/−^* (Tg/*Tace^−/−^*) mice.

A mouse *Tace* cDNA with an HA-epitope (YPYDVPDYA) sequence added to the 3′-teminus was generated using PCR-based methods and subcloned into the pCAGGS vector. HA-epitope tagged-TACE has been shown to exhibit comparable shedding activity towards its substrates to non-tagged wild-type TACE [22], [30]. A transgene fragment containing the CAG promoter, *Tace* cDNA, HA epitope, and polyadenylation signal sequence (Fig. 1A) was injected into fertilized zygotes obtained from superovulated donor mice. Several founder mice were obtained, and two independent lines were selected for further analysis based on the expression levels of the transgene. The phenotypes of these two transgenic lines (line-004 and -005) were nearly identical, and the data obtained using the line-004 are presented in this study. A Southern blot was performed using genomic DNA collected from the tail to confirm a single integration site of the transgene and to determine the copy number of the integration. All of the animal experiments in this study were approved by the Institutional Animal Care and Use Committee of the Keio University, School of Medicine (Permit Number: 09101).

### Histology

The tissues were fixed in 4% paraformaldehyde/PBS, embedded in paraffin, sectioned, and stained with hematoxylin and eosin. The sections were photographed using a DXM1200 camera (Nikon, Tokyo, Japan) and a BX50 microscope (Olympus, Tokyo, Japan).

### Quantitative RT-PCR

RNA was extracted from the tissues or cultured cells using Sepasol RNA I Super G (Nacalai Tesque, Kyoto, Japan) and reverse-transcribed using ReverTra Ace (Toyobo, Osaka, Japan). The PCR amplification and quantification were performed using SYBR premix ExTaqII (Takara Bio, Shiga, Japan) and LightCyclerII (Roche). The relative mRNA expression levels were normalized to the expression level of the β-Actin transcripts. The sequences of the oligonucleotides used in this study will be provided upon request.

### Western blotting

The tissues and cells were lysed in lysis buffer (1% Triton X-100, 150 mM NaCl, 0.5 mM EDTA, 10 mM Tris-HCl (pH 7.4), 1 mM 1,10-phenanthroline, and protein inhibitor cocktail (Sigma-Aldrich)), and the lysed samples were separated on SDS-polyacrylamide gels and transferred to nitrocellulose membranes. The anti-sera against the cytoplasmic domain of mouse TACE was produced as previously described [31]. The anti-HA-epitope antibody (clone; 3F10), anti-ADAM10, and anti-β-Actin antibody were purchased from Roche, Calbiochem, and Sigma-Aldrich, respectively.

### Shedding assay

Mouse embryonic fibroblasts (mEFs) collected from E13.5 *Tace*-Tg embryos were immortalized with the SV40 large T antigen, as previously described [32]. The cells were transfected with alkaline phosphatase (AP)-tagged TGFα using Fugene HD (Roche), as previously described [22]. Fresh Opti-MEM (Invitrogen) medium with or without the indicated reagents (PMA and/or GM6001) was added 24 h after the transfection and incubated for an hour. The AP activity was measured by colorimetry, as previously described [33], [34].

### Cell surface labeling

The cells were washed twice in ice-cold PBS, and the cell surface molecules were labeled using Pierce Cell Surface Protein Isolation Kit (Thermo) according to the manufacturer's instructions, with some modifications. The labeled samples were affinity precipitated with neutravidin beads overnight at 4°C, and the affinity precipitated material was separated by SDS-PAGE and analyzed by Western blotting.

### Septic shock

Septic shock was induced by the intraperitoneal injection of lipopolysaccharide (LPS, 5 μg) and D-galactosamine (20 mg). The mice were closely monitored until the mortality was 100%. For the analysis of the serum cytokines, the sera were collected at 3 h after the treatment in a separate experiment. The serum levels of TNFα, TNFR1, TNFR2, and CD62L were analyzed by sandwich ELISA (Quantikine; R&D Systems) according to the manufacturer's instructions.

### Statistical analysis

The Student's *t*-test for two samples, assuming equal variances, was used to calculate the *p* values. The statistical analyses were performed using Prism 5 (GraphPad software), and *p* values smaller than 0.05 were considered statistically significant. All of the experiments were conducted in triplicate.

## Results and Discussion

### Mice systemically overexpressing TACE exhibit no overt defects

To understand how the enhanced expression of *Tace* transcripts would affect the TACE activity *in vivo*, we generated transgenic mice that expressed an HA-tagged TACE driven by a CAG promoter (Fig. 1A). The HA-epitope was added to the C-terminus of the cytoplasmic domain of TACE to facilitate the detection of the protein; an HA-tagged TACE expression vector used in previous studies showed no interference on the activity of TACE *in vitro* [22]. The *Tace*-transgenic mice (henceforth referred to as *Tace*-Tg mice) were viable and fertile and did not show any overt developmental defects (Fig. 1B). Matings between the wild-type control mice and *Tace*-Tg mice yielded offspring in the Mendelian ratio (wild-type mice, 186; *Tace*-Tg mice, 201; χ^2^ = 0.446).

We first evaluated the expression levels of the *Tace* transcripts in the *Tace*-Tg mice by quantitative RT-PCR. As shown in Figure 1C, a significant increase in the transcript levels of *Tace* was observed in all the tissues examined, including the liver, lung, skin, spleen, bone marrow (BM), and thymus. The relative increase was lower (approx. 4–6 times) in the immune organs (spleen, BM, and thymus) and higher (approx. 30–300 times) in the other tissues, indicating that the basal levels of *Tace* transcripts and/or CAG promoter activity differ across these tissues. The increased expression of the TACE protein in these tissues was also confirmed using an anti-HA antibody (which specifically binds to the transgenically introduced HA-tagged TACE) and an anti-TACE antibody (which recognizes both endogenous and transgenically introduced TACE) (Fig. 1D). Necrotomy of 8-week-old *Tace*-Tg and control littermate mice showed no apparent defects at either the macroscopic or histological level (Fig. 1E). These observations indicate that a highly enhanced expression level of TACE does not have a profound impact on normal development or homeostasis. To confirm that the transgenically introduced TACE was fully functional *in vivo*, we mated the *Tace*-Tg mice with *Tace^+/−^* mice [32] to generate *Tace*-Tg*/Tace^−/−^* mice. The *Tace*-Tg*/Tace^−/−^* mice expressed no endogenous TACE protein, only expressing the transgenic HA-tagged TACE under the control of the CAG promoter. These mutant mice were also viable and revealed no apparent defects at either the macroscopic or histological level (Fig. 1E and data not shown). We, therefore, concluded that transgenic HA-tagged TACE was fully functional *in vivo* and that the transcriptional regulation of *Tace* by its endogenous promoter is not necessarily essential for the functional regulation of TACE during development and postnatal growth.

### Unaltered shedding activity in *Tace*-Tg-derived mEFs

**Figure 2:**
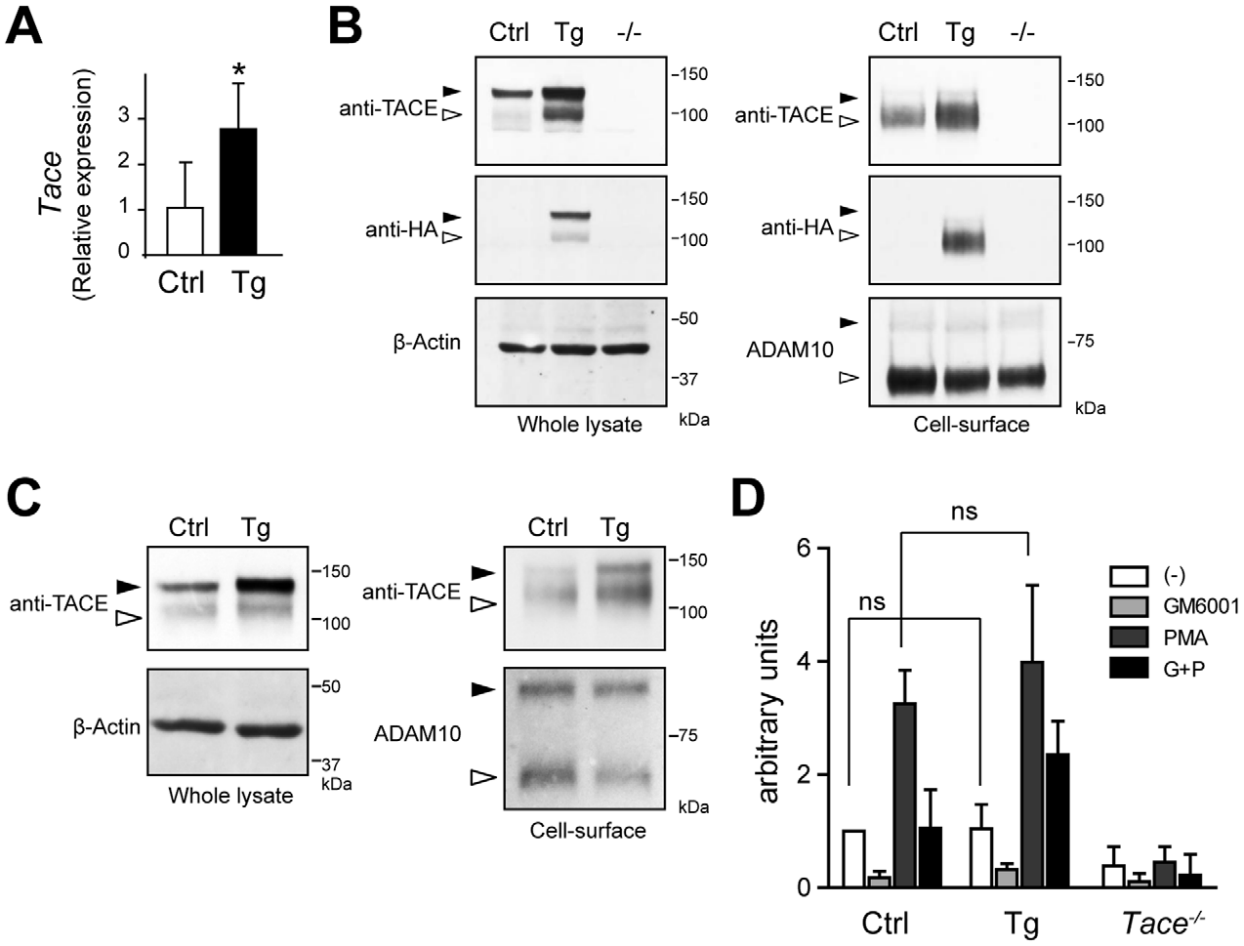
An increase in the mature TACE in *Tace*-Tg mEFs does not significantly affect the shedding properties of TGFα. (A) Quantitative RT-PCR analysis of *Tace* expression in the control (Ctrl) and *Tace*-Tg (Tg)-derived mEFs. (B, C) Cell surface molecules in mEFs (B) derived from control (Ctrl), *Tace*-Tg (Tg), and *Tace^−/−^* (−/−) mice, and splenocytes (C) derived from control (Ctrl) and *Tace*-Tg (Tg) were biotin-labeled and analyzed by Western blotting before (Whole lysate) and after (Cell-surface) affinity precipitation with neutravidin beads. The membranes were reprobed with anti-β-Actin antibody and anti-ADAM10 antibody to serve as loading controls for total protein from the whole cell lysate and the cell-surface protein, respectively. Black arrowheads, pro-form. White arrowheads, mature form. Please note that there are small amount of biotinylated pro-form TACE and ADAM10 in panel (C) that were labeled through leakage of the biotin reagent during the procedure. (D) Evaluation of TGFα shedding in the control (Ctrl), *Tace*-Tg (Tg), and *Tace^−/−^* mEFs by a colorimetric assay. Bars, S.D. *p<0.05. ns, not significant.

To confirm that the introduction of transgenic HA-tagged-TACE resulted in an increase in the total amount of the mature protein, we examined the expression level of mature TACE by labeling the proteins expressed on the cell surface with membrane-impermeable biotin. The TACE protein is initially produced as a pro-form and is cleaved by a furin protease before it is expressed as a proteolytically active mature form on the cell surface [21]. Therefore, only the mature form could be labeled with a membrane-impermeable biotin. We first confirmed the increase in the *Tace* transcript levels in the mEFs derived from the *Tace*-Tg mice (Fig. 2A). As shown in Figure 2B (left panels), a Western blot of the cell lysates using an anti-TACE antibody revealed an increase in the amount of both the pro-form (black arrowhead) and mature form (white arrowheads) in the *Tace*-Tg mEFs. The expression and maturation of the HA-tagged TACE were also confirmed by Western blotting using an anti-HA antibody (Fig. 2B, left panels). The lysates were affinity precipitated using neutravidin-conjugated beads and detected with anti-TACE and anti-HA antibodies. Consistent with the results of the Western blots of the cell lysates, there was a sharp increase in the amount of the biotin-labeled mature form in the *Tace*-Tg mEFs (Fig. 2B, right panels). These observations show that the *Tace*-Tg mEFs express more mature TACE than the control mEFs, and that the maturation and trafficking of the exogenous HA-tagged TACE to cell surface are not hampered in the *Tace*-Tg mice. The increase in the amount of mature TACE was also confirmed in the *Tace*-Tg splenocytes (Fig. 2C).

The apparent lack of developmental defects in the *Tace*-Tg mice indicates that the increase in the *Tace* transcript levels does not significantly affect the shedding of its substrates. To test this hypothesis, we performed an *in vitro* shedding assay using AP-tagged TGFα, as previously described [22]. We introduced an AP-TGFα expression vector into immortalized mEFs and indirectly evaluated the shedding activity by measuring the AP activity released in the supernatant using colorimetry [34]. TGFα is a well-established TACE substrate, and the cleavage of the membrane-bound pro-TGFα can be significantly stimulated *in vitro* with phorbol esters, such as phorbol 12-myristate 13-acetate (PMA). As shown in Figure 2D, we found no significant difference in the shedding profile of AP-TGFα between the mEFs derived from the control and *Tace*-Tg embryos. The shedding activity was similarly enhanced upon PMA stimulation and suppressed by a broad-range metalloprotease inhibitor, GM6001. These observations indicate that the excess TACE protein in the *Tace*-Tg-derived mEFs does not significantly affect the overall shedding activity, at least under the present experimental conditions.

### LPS-induced production of soluble TNFα is comparable between control and *Tace*-Tg mice

**Figure 3:**
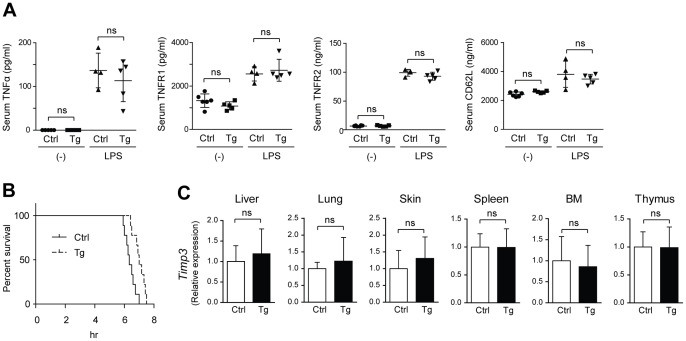
No apparent change in the serum levels of TACE substrates was observed in *Tace*-Tg mice. (A) The serum levels of TNFα, TNFR1, TNFR2, and CD62L were analyzed by ELISA in control and *Tace*-Tg (Tg) mice. The sera were collected under unchallenged conditions (−) or 3 h after the intraperitoneal injection of LPS and D-galactosamine. (B) Survival curve of the control (Ctrl) and *Tace*-Tg (Tg) mice treated with LPS and D-galactosamine (*n* = 18). (C) Quantitative RT-PCR of *Timp3* in the liver, lung, skin, spleen, bone marrow cells (BM), and thymus collected from the control (Ctrl) and *Tace*-Tg mice. Bars, S.D. ns, not significant.

Several studies have shown a causal relationship between the enhanced expression of TACE and cancer progression and inflammatory disease [10], [11], [35], and these studies have indicated that the upregulation of TACE leads to an enhanced shedding of its substrates and, consequently, a more aggressive phenotype. Conversely, these observations also suggest that the effect of the increased expression of TACE could be manifested under pathological conditions. To test this hypothesis, we next evaluated the production of soluble TNFα in serum using a murine model of endotoxin shock. The control and *Tace*-Tg mice were intraperitoneally injected with LPS to induce TNFα production; LPS is a major component of the cell membrane of Gram-negative bacteria, and it elicits a strong immune response in mammalian immune cells. Because LPS can stimulate the production of TNFα in immune cells, we hypothesized that the amount of soluble TNFα released into the supernatant would show a positive correlation with the amount of TACE expressed in the immune cells. However, contrary to our expectation, there was no difference in the serum levels of TNFα or any of the TACE substrates examined (TNFα receptor 1, TNFα receptor 2, and CD62L) between the control and *Tace*-Tg mice under unchallenged conditions or after LPS treatment (Fig. 3A). Furthermore, we also found that the *Tace*-Tg mice were not more susceptible to LPS-induced septic shock than the control animals; in fact, they were slightly more resistant (Fig. 3B).

Given these observations, we next examined whether there was a change in the expression levels of TIMP3 in the *Tace*-Tg mice-derived cells and tissues. TIMP3 is a critical regulator of TACE, and *Timp3^−/−^* mice were shown to exhibit an overt immune response due to the overproduction of soluble TNFα [36], [37]. Furthermore, it has recently been shown that TIMP3 suppresses TACE activity by directly binding to TACE dimers and that MAP kinase activation stimulates TACE activity by suppressing the dimerization of TACE and the binding of TIMP3 to TACE [29]. Therefore, in theory, overt TACE activity can be offset if the expression of TIMP3 correlates with that of TACE. However, we did not find any difference between the control and *Tace*-Tg mice with regard to the transcription levels of *Timp3* in any of the organs examined (Fig. 3C). These observations indicate that even though TIMP3 is certainly a critical regulator of TACE, the activity of TACE cannot be simply deduced from the ratio between the TIMP3 and TACE expression levels. In fact, although *Timp3^−/−^* mice have been shown to develop hepatic inflammation due to increased TACE-TNFα activity [36], we did not observe any defects in the *Tace*-Tg liver, even though approximately 300 times more *Tace* transcript was expressed in the *Tace*-Tg *versus* the control liver (Fig. 1C).

The mechanisms underlying the activation of TACE remain controversial. It is clear that the cleavage of the prodomain is necessary for TACE maturation, and recent studies have revealed that immune cells lacking iRhom2 (RHBDF2), a proteolytically inactive member of the rhomboid protease family, are defective for this process and incapable of releasing soluble TNFα [38], [39]. The present study indicates that although the conversion of TACE from the pro- to mature form is critical for TACE to become functional, the amount of mature TACE does not directly correlate with the overall shedding activity. Therefore, it may be that cells require only a certain amount of TACE and that any excess TACE above the threshold level does not significantly contribute to the overall shedding activity in a given cell or tissue. In agreement with this hypothesis, the present study also suggests that the transcription of *Tace* does not have to be rigorously regulated and can even be highly enhanced without altering its functions *in vivo*, at least during normal development and under physiological conditions, as illustrated by the apparently normal phenotype observed in the *Tace*-Tg/*Tace^−^* ^/*−*^ mice (Fig. 1E). Furthermore, these observations may indicate a model whereby the amount of mature TACE is sequentially regulated at transcriptional and post-translational levels. In this model, the above mentioned iRhom2 in immune cells [38], [39] or an equivalent molecule(s) in non-immune cells functions, independently from the transcriptional regulation, as a gatekeeper to control the amount of TACE protein exiting the endoplasmic reticulum. An apparent increase in the amount of pro-form TACE in *Tace*-Tg mice tissues may support this tentative model (Fig. 1D).

In summary, this study demonstrates the lack of a direct correlation between the TACE expression levels and the shedding of TACE substrates *in vivo*. The observation that the amount of cleaved molecules in the serum remained static, irrespective of the TACE expression levels (Fig. 3A), may suggest that the time-limiting factor for the proteolysis of membrane-anchored proteins is dependent on the amount of the target substrates but not that of TACE itself. Alternatively, it is also possible that the ectodomain shedding by TACE occurs only in a certain micro-niche on the cell surface, excluding any supernumerary TACE from participating in the cleavage of the substrate. Nevertheless, the contributions of increased TACE expression to the pathogenesis of cancers and inflammatory diseases should be interpreted cautiously because an increase in TACE expression may not necessarily indicate the enhanced shedding activity of TACE in these disorders.

### Addendum

During the revision of the manuscript, an independent study [40] presented data in agreement with our results that systemic overexpression of TACE *in vivo* does not lead to overt defects.
